# Mitochondria and Quality Control Defects in a Mouse Model of Gaucher Disease—Links to Parkinson’s Disease

**DOI:** 10.1016/j.cmet.2013.04.014

**Published:** 2013-06-04

**Authors:** Laura D. Osellame, Ahad A. Rahim, Iain P. Hargreaves, Matthew E. Gegg, Angela Richard-Londt, Sebastian Brandner, Simon N. Waddington, Anthony H.V. Schapira, Michael R. Duchen

**Affiliations:** 1 Department of Cell and Developmental Biology, University College London, London WC1E 6BT, UK; 2 UCL Consortium for Mitochondrial Research, University College London, London WC1E 6BT, UK; 3 Gene Transfer Technology Group, Institute for Women’s Health, University College London, London WC1E 6BT, UK; 4 Department of Molecular Neuroscience, Institute of Neurology, University College London, London WC1N 3BG, UK; 5 Division of Neuropathology and Department of Neurodegenerative Diseases, Institute of Neurology, University College London, London WC1N 3BG, UK; 6 Department of Clinical Neurosciences, UCL Institute of Neurology, Royal Free Campus, London NW3 2PF, UK; 7 School of Pathology, University of the Witwatersrand, Johannesburg 2000, South Africa

## Abstract

Mutations in the *glucocerebrosidase* (*gba*) gene cause Gaucher disease (GD), the most common lysosomal storage disorder, and increase susceptibility to Parkinson’s disease (PD). While the clinical and pathological features of idiopathic PD and PD related to *gba* (PD-GBA) mutations are very similar, cellular mechanisms underlying neurodegeneration in each are unclear. Using a mouse model of neuronopathic GD, we show that autophagic machinery and proteasomal machinery are defective in neurons and astrocytes lacking *gba*. Markers of neurodegeneration—p62/SQSTM1, ubiquitinated proteins, and insoluble α-synuclein—accumulate. Mitochondria were dysfunctional and fragmented, with impaired respiration, reduced respiratory chain complex activities, and a decreased potential maintained by reversal of the ATP synthase. Thus a primary lysosomal defect causes accumulation of dysfunctional mitochondria as a result of impaired autophagy and dysfunctional proteasomal pathways. These data provide conclusive evidence for mitochondrial dysfunction in GD and provide insight into the pathogenesis of PD and PD-GBA.

## Highlights

• Autophagic and proteasomal pathways are suppressed in *gba* knockout mice
• α-Synuclein accumulates and forms deposits in *gba* knockout mouse brainstem
• Neurons and astrocytes from *gba* knockout mice harbor dysfunctional mitochondria
• Mitochondria do not recruit Parkin and accumulate in *gba* knockout neurons

## Graphical Abstract

**Figure:**
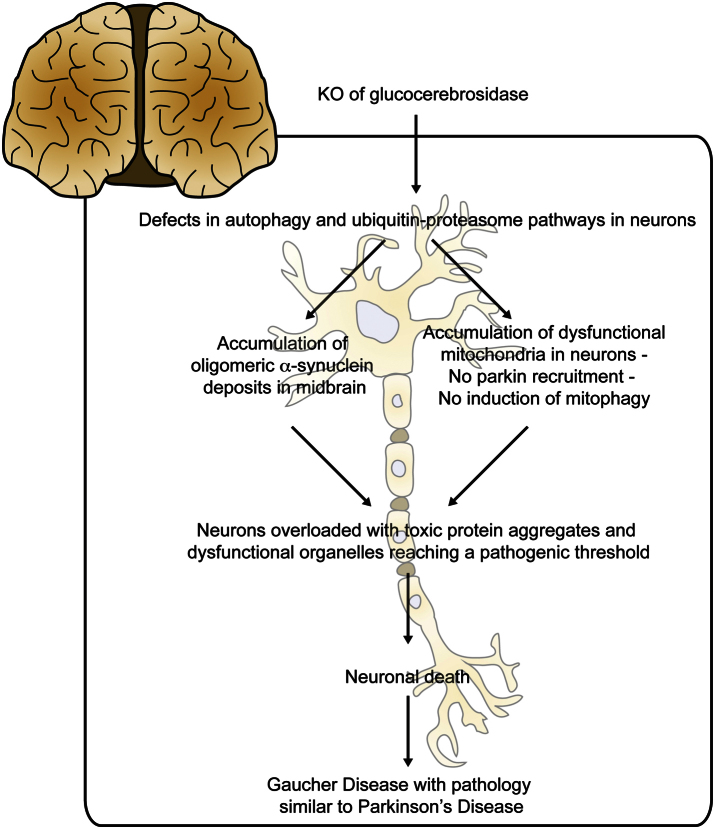

## Introduction

Lysosomal storage disorders (LSDs), of which Gaucher disease (GD) is the most common, are rare inherited metabolic diseases. GD is caused by mutations in the *glucocerebrosidase* (*gba*) gene resulting in decreased enzyme (GCase) activity, accumulation of substrate (glucocerebroside) within lysosomes, and compromised lysosomal activity (Brady et al., 1965). GCase is a lysosomal enzyme responsible for the conversion of glucocerebroside to glucose and ceramide. GD is classified into three clinical subsets based on age and onset of neurological involvement, although the relationship between phenotype and genotype remains obscure (Grabowski, 2008). Type I (OMIM#230800) is the most common and is classified according to the absence of central nervous system (CNS) abnormalities. Type II (OMIM#230900) and III (OMIM#2301000) are less prevalent. Type II exhibits rapid onset severe and progressive neurodegeneration, while CNS involvement in type III is chronic and less severe.

The pathological and neurological symptoms displayed by type I GD patients share many features of Parkinson’s disease (PD), including Lewy body formation, loss of dopamingeric neurons, bradykinesia, rigidity, and rest tremors (Tayebi et al., 2001). Indeed, Parkinsonism has been reported in a subset of GD patients and carriers, suggesting a mechanistic link between the two disorders (Westbroek et al., 2011). In addition, several large multicenter genetic studies have shown the incidence of *gba* mutations is increased among patients with PD (Anheim et al., 2012; Lesage et al., 2011; Neumann et al., 2009). Of all the genetic predisposing factors thus far identified for PD, *gba* are numerically the most important (Mata et al., 2008; Mitsui et al., 2009; Sidransky et al., 2009). Postmortem study of PD patient brains with *gba* mutations revealed extensive GCase deficiency with the substantia nigra most affected (Gegg et al., 2012). Recent reports have detailed the involvement of an α-synuclein feedback loop in GD and synucleinopathies (Gegg et al., 2012; Mazzulli et al., 2011). Mazzulli et al. (2011) demonstrated that knockdown of *gba* contributed to toxic buildup of insoluble α-synuclein within lysosomes, compromising lysosomal protein degradation.

Reduced lysosomal activity has far-reaching implications for autophagy, as the fusion of lysosomes with autophagosomes is a critical step in the degradation of redundant or damaged cellular material. Despite the essential role of lysosomes in organelle turnover and the fact that defects in mitochondrial quality control are strongly implicated in PD, nothing is known about the fate of mitochondria in GD. Accumulating evidence indicates that defects in mitochondrial function are particularly potent in postmitotic cells such as neurons, which show an absolute requirement for mitochondrial oxidative phosphorylation to maintain local ATP levels and to buffer calcium signals (Chan, 2006). This is most important during cellular metabolic stress, as neurons have a limited capacity to switch metabolism from oxidative phosphorylation to glycolysis (Bolaños et al., 2010), placing absolute requirements on mitochondrial function and positioning. As such, mitochondrial activity, shape, and movement are intimately tied with the functional status of the cell itself (Osellame et al., 2012).

Here we show that defects in both autophagic and proteasomal pathways in a type II GD mouse model lead to accumulation of fragmented and bioenergetically compromised mitochondria in primary neurons and astrocytes lacking *gba*. Common hallmarks of neurodegeneration, including accumulation of ubiquitinated proteins and α-synuclein deposits found in PD, are also seen here in GD mouse brains. The work presented here therefore suggests that similar mechanisms of neurodegeneration underlie the pathogenesis of both GD and PD, explaining the strong genetic linkage and analogous pathologies.

## Results

### Autophagic and Ubiquitin-Proteasome Pathways Are Defective in *gba*^−/−^ Neurons

Many LSDs have been reported to harbor defects in autophagy (Elrick et al., 2012; Jennings et al., 2006; Settembre et al., 2008a). Although GD has been classified since the 1950s as an inborn error of metabolism, very little is known as to how loss of GCase activity impairs the autophagic component of the pathway. The type II GD mouse model employed in this study was generated by Enquist et al. (2007) (see Figure S1A online). Complete *gba* knockout is lethal at postnatal day 15 (Enquist et al., 2007). Preliminary measurements were performed to confirm that this model showed loss of GCase activity and protein in the brain. Mixed cultures of cortical neurons and astrocytes were screened for GCase activity postgenotyping using liver cDNA (Figures S1B and S1C). Heterozygote cells (*gba*^*+/−*^) exhibited a 42.7% decrease in GCase activity compared to wild-type (*gba*^*+/+*^) (Figure S1C and Table S1). Mouse *gba*^*−/−*^ cells retained residual GCase activity (Figure S1C and Table S1). This was reflected in midbrain GCase protein levels following immunoblotting while *gba*^*+/−*^ levels were intermediate with *gba*^*+/+*^ (Figure S1D).

**Figure 1:**
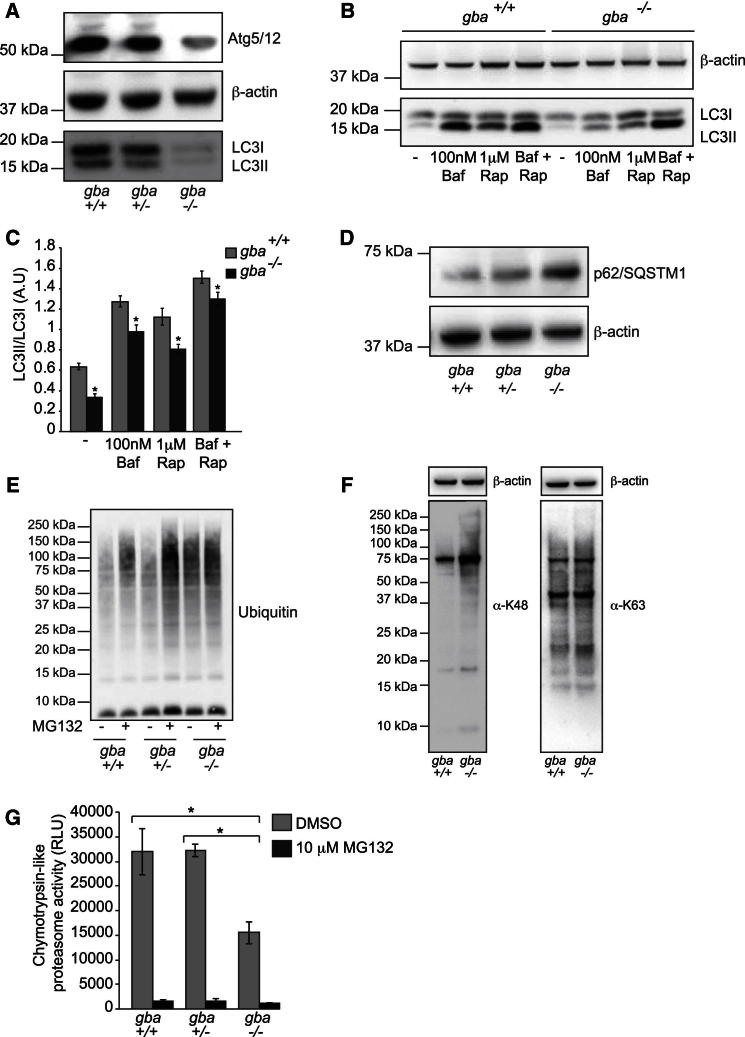
Loss of *gba* Impairs Both Autophagic and Proteasomal Degradation Machinery (A) Autophagic markers from mixed midbrain *gba*^*+/+*^, *gba*^*+/−*^, and *gba*^*−/−*^ cultures were analyzed via western blotting using antibodies as indicated. (B) Autophagic flux was analyzed in *gba*^*+/+*^ and *gba*^*−/−*^ midbrain neurons. Neurons were treated with 100 nM bafilomycin A1 and 1 μM rapamycin and flux assayed by comparing LC3I and LC3II levels via western blotting. β-actin was used as a loading control. (C) Densitometry analyzes of autophagy of (B) expressed as a ratio of LC3II/LC3I. Error bars, ± SEM. ^*^p < 0.05. (D) Accumulation of p62/SQSTM1 was analyzed via immunoblotting. β-actin was used as a loading control. (E) Midbrain neurons were treated with either 10 μM MG132 or vehicle control DMSO. Proteins were extracted and analyzed via western blotting and stained with anti-ubiquitin antibodies. (F) Ubiquitin linkage of isolated midbrain analyzed using western blotting with antibodies as indicated. β-actin was used as a loading control. (G) Proteasomal activity (chymotrypsin activity) was measured using the Proteasome Glo assay where activity is proportional to the released luciferase. RFU, relative luciferase units. Data represent the mean ± SEM (n = 3, each experiment contained triplicates for each genotype). ^*^p < 0.05.

We then analyzed the integrity of the macroautophagy pathway. Immunoblot analysis shows a decrease in LC3I as well as LC3II (a marker of autophagosomes) in *gba*^*−/−*^ cells in comparison to controls (Figure 1A). To further confirm defects upstream of lysosomal involvement, expression of Atg5/12 was analyzed. As with LC3II, expression of conjugated Atg5/12 in *gba*^*−/−*^ cells was also reduced, implying impaired regulation of LC3 conjugation with phosphatidylethanolamine. (Figure 1A). Downregulation of Atg5/12 does not appear to occur at transcript level, as Atg5 mRNA derived from *gba*^*+/+*^, *gba*^*+/−*^, and *gba*^*−/−*^ cDNA were not significantly different when analyzed using qPCR (Figure S2A). In order to define the flux through the macroautophagic pathway, isolated midbrain neurons were treated with 100 nM bafilomycin A1, 1 μM rapamycin, or both drugs in combination for 6 hr (Figure 1B). In control neurons, bafilomycin increased levels of LC3II species as expected, as autophagy is blocked after autophagosome formation and before the autophagosome can fuse with the lysosome. Rapamycin increased both species of LC3, indicating increased autophagy, expressed as a ratio of LC3II/LC3I and LC3II/β-actin (Figures 1B and 1C and Figure S2B). Bafilomycin caused a small but significant increase in LC3II species in *gba*^*−/−*^ neurons, suggesting that the block in autophagy in GD may not be complete (Figures 1B and 1C). In addition, LC3I/II levels were analyzed in response to starvation (Earle’s balanced salt solution minus amino acids and serum), a more physiologically relevant treatment than rapamycin (Figure S2C). Similarly, both species of LC3 were increased in *gba*^*−/−*^ cells treated with rapamycin and starvation, suggesting that the autophagic machinery in type II GD does retain the ability to upregulate the pathway, although not to the same extent as *gba*^*+/−*^ cells (Figures 1B and 1C and Figure S2C). It does, however, appear that under physiological disease conditions compensatory mechanisms are not active, and that the autophagy pathway is severely compromised.

Although macroautophagy is the major pathway for organelle turnover, it is not the only pathway through which cellular material is degraded; the ubquitin-proteasome system (UPS) contributes to cellular quality control by degrading damaged and misfolded proteins. p62, also called sequestosome 1 (SQSTM1), links the two pathways; it binds to ubiquitinated substrates and LC3, thereby promoting autophagic degradation of otherwise proteasomal substrates. Western blots of midbrain homogenates showed accumulation of p62 in *gba*^*−/−*^ (and not *gba*^*+/+*^ or *gba*^*+/−*^) cells, implying that in addition to the defect in autophagy, the UPS may be consequently impaired (Figure 1D). More specifically, p62 accumulated in midbrain *gba*^*−/−*^ neurons and astrocytes (detected by immunofluorescence, Figure S2D). p62/SQSTM1 levels were also analyzed in response to starvation, in which the buildup of accumulated p62/SQSTM1 in *gba*^*−/−*^ cells, detected using immunoblotting, was slightly alleviated by induction of autophagy (Figure S2C). Buildup of ubiquitin (Ub) was analyzed using MG132 treatments and western blotting. MG132 treatment impaired Ub-tagged protein turnover in *gba*^*+/+*^, *gba*^*+/−*^, and *gba*^*−/−*^ cells compared to the control (Figure 1E). Difference in Ub staining between the control and MG132 treated *gba*^*−/−*^ samples was minimal, suggesting that the proteasomal degradation machinery in this system may already be inhibited prior to chemical treatment. Ub linkage was analyzed via western blotting, with the majority of accumulated Ub being K-48 derived (destined for the proteasome) rather than K-63 (autophagic degradation) (Figure 1F).

To confirm loss of proteasomal activity in the *gba*^*−/−*^ mice, the ability of the proteasome to degrade a fluorescently tagged substrate (Suc-LLVY-aminoluciferin) was analyzed. Using the Proteasome Glo assay to monitor the chymotrypsin-like activity of the proteasome, neurons were treated with either 10 μM MG132 (as a positive control for loss of proteasome activity) or DMSO. The proteasome activity of the *gba*^*−/−*^ neurons is significantly less than that of both the *gba*^*+/+*^ and *gba*^*+/−*^, as shown by the reduced amount of luciferase released (Figure 1G). Taken together, these data suggest in addition to defective autophagy in neurons lacking *gba*, the UPS degradation pathway might also be affected.

### Defective Proteostasis Affects α-Synuclein Turnover

**Figure 2:**
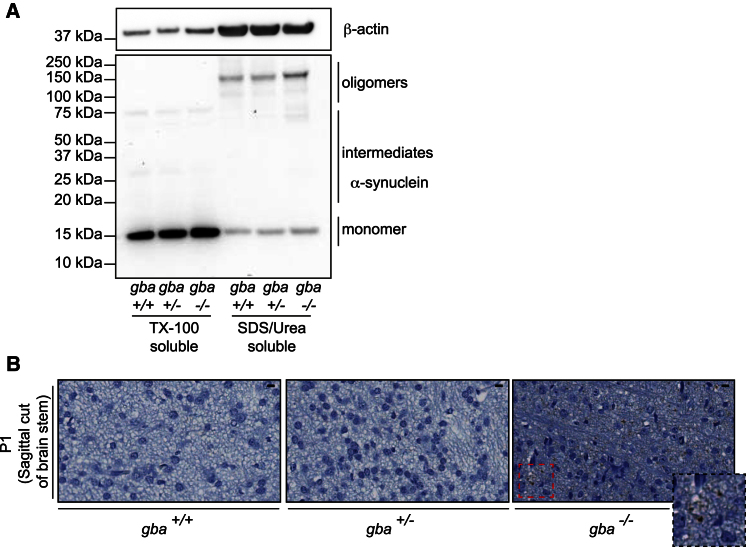
Accumulation of α-Synuclein as a Consequence of Impaired Quality Control (A) Levels of soluble and insoluble α-synuclein from midbrain of P1 *gba*^*+/+*^ and *gba*^*−/−*^ mice. TX-100 soluble and insoluble (SDS/Urea) factions were isolated and analyzed via western blotting with α-synuclein antibodies. β-actin was used as a loading control. (B) Sagittal brain stem sections from *gba*^*+/+*^, *gba*^*+/−*^, and *gba*^*−/−*^ mice were stained with α-synuclein antibodies. Scale bar, 1,000 μm.

α-Synuclein and Lewy body formation are hallmarks of both GD and PD-GBA. We next investigated whether the mouse brain showed similar pathology to that of the postmortem human brain (Tayebi et al., 2003). Monomeric, intermediate, and oligomeric species of α-synuclein (from isolated midbrain) were fractionated via differential detergent treatments and sonication and separated by electrophoresis, which revealed an increase in α-synuclein oligomeric species in the *gba*^*−/−*^ midbrain compared with *gba*^*+/+*^ and *gba*^*+/−*^ (Figure 2A). In normal cellular homeostasis, α-synuclein is trafficked via chaperone-mediated autophagy (CMA) and ultimately degraded by lysosomes (Cuervo et al., 2004). Given the defects observed in this pathway, we hypothesized an inability of these neurons to turn over the aggregated protein. In sagittal sections of the brain stem in *gba*^*+/+*^, *gba*^*+/−*^, and *gba*^*−/−*^ P1 mice, α-synuclein deposits were identified only in *gba*^*−/−*^ brain stem sections (Figure 2B). This confirms that loss of GCase activity and consequent defects in autophagy impair α-synuclein turnover.

### Mitochondrial Function Is Impaired in Neurons and Astrocytes in Type II GD

**Figure 3:**
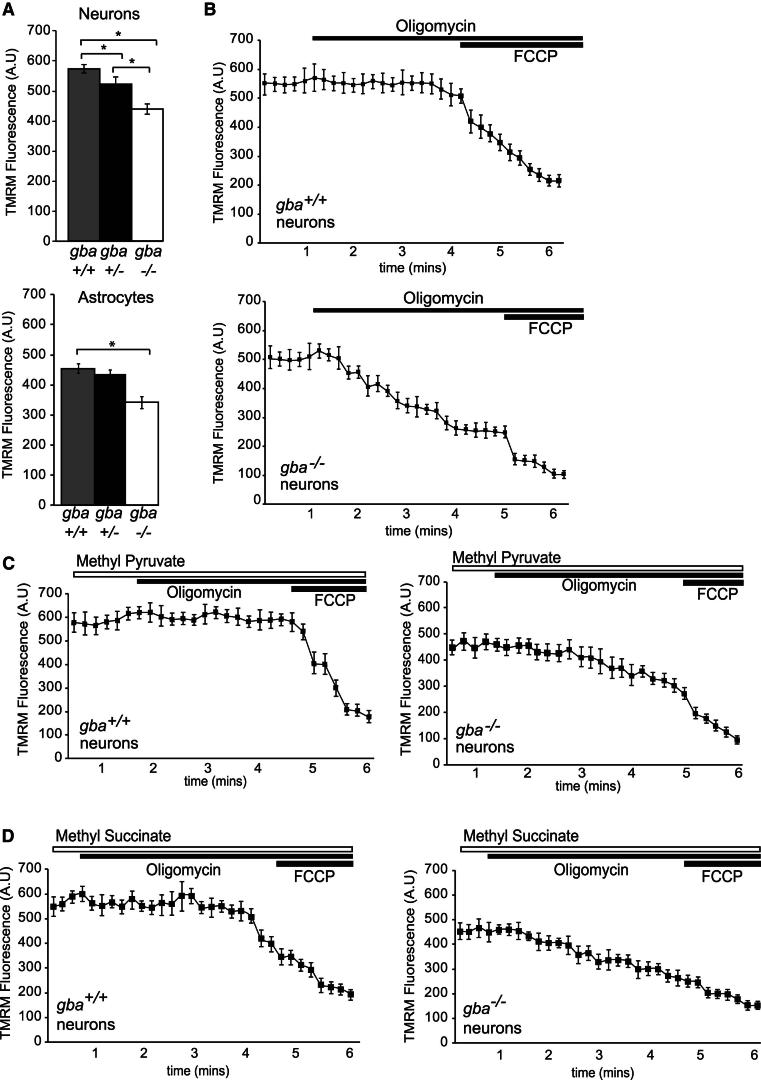
Mitochondrial Physiology Is Affected in *gba*^*−/−*^ Neurons and Astrocytes (A) Neurons and astrocytes from *gba*^*+/+*^, *gba*^*+/−*^, and *gba*^*−/−*^ mice were stained with TMRM. The mean florescence intensity in mitochondria was analyzed via confocal microscopy (n = 3, >32 cells analyzed/experiment). (B) Neurons from *gba*^*+/+*^ and *gba*^*−/−*^ bathed in TMRM containing recording solution and fluorescence intensity analyzed via confocal microscopy. After 1 min, 1 μM oligomycin was added followed by 1 μM FCCP (n = 5, three cells analyzed/experiment). (C) Neurons from *gba*^*+/+*^ and *gba*^*−/−*^ incubated with 5 mM methyl pyruvate 5 min prior to imaging. Cells bathed in 25 nM TMRM and 5 mM methyl pyruvate containing recording solution and fluorescence intensity analyzed via confocal microscopy. After 90 s, 1 μM oligomycin was added, followed by 1 μM FCCP (n = 3, three cells analyzed/experiment). (D) As in (C), except cells were bathed in 10 mM methyl succinate, (n = 3, four cells/experiment). All data in this figure represent the mean ± SEM, ^*^p < 0.05.

Mitochondrial dysfunction has been widely implicated in familial PD, with mutations in PINK1, Parkin, DJ-1, and HtrA2/Omi resulting in defects in mitochondrial dynamics, physiology, and quality control (Bonifati et al., 2003; Narendra et al., 2008; Plun-Favreau et al., 2007; Valente et al., 2004). Little is known about mitochondrial status in GD or about the contribution of mitochondrial dysfunction to its pathogenesis. Given the dependence of neurons on mitochondrial activity, we sought to characterize how suppressed quality control pathways affected mitochondrial function. In order to establish whether defective organelle turnover in *gba*^*−/−*^ cells had a direct impact on mitochondrial volume in cells, we assayed mitochondrial mass using confocal microscopy. Cells were stained with calcein-AM (to measure cytosolic volume) and MitoTracker Red, and mitochondrial volume fraction calculated from binarized images (Figure S3A). Mitochondrial volume occupancy was increased in *gba*^*−/−*^ cells compared to *gba*^*+/+*^ and *gba*^*+/−*^ cells, consistent with a reduced organelle turnover in *gba*^*−/−*^ cells. Mitochondrial membrane potential (ΔΨ_m_) was measured using TMRM and quantitative confocal imaging. The resting ΔΨ_m_ of the *gba*^*−/−*^ neurons and astrocytes was significantly lower than that of cells derived from *gba*^*+/+*^ or *gba*^*+/−*^ mice (Figure 3A). To determine the functionality of the respiratory chain, *gba*^*+/+*^ and *gba*^*−/−*^ neurons were incubated with oligomycin (Figure 3A), as reversal of the F_1_F_o_-ATPase has been implicated in the PINK1 knockout associated with PD (Gandhi et al., 2009). Oligomycin had no effect on ΔΨ_m_ in *gba*^*+/+*^ neurons but caused progressive dissipation of ΔΨ_m_ in *gba*^*−/−*^ neurons, showing that the reduced ΔΨ_m_ was maintained by reversal of the ATP synthase at the expense of ATP. These observations strongly suggest an impaired respiratory chain or impaired substrate supply. To differentiate between these, cells were incubated with methyl pyruvate and methyl succinate (Figures 3C and 3D), neither of which were able rescue ΔΨ_m_ in *gba*^*−/−*^ neurons (Figures 3C and 3D), pointing to an impaired respiratory chain as a cause of the mitochondrial dysfunction in these cells.

### Respiratory Chain Defects in *gba*^*−/−*^ Mice

**Figure 4:**
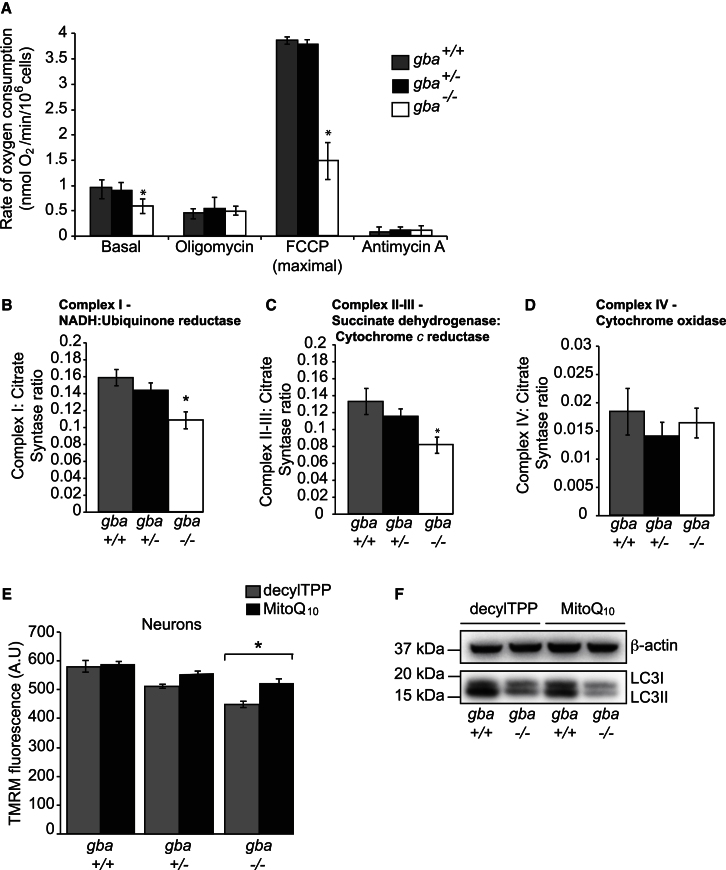
*gba*^*−/−*^ Mitochondria Have a Reduced Respiratory Chain Activity (A) Oxygen consumption rates of *gba*^*+/+*^, *gba*^*+/−*^, and *gba*^*−/−*^ mixed midbrain cultures. Basal oxygen consumption was measured over 3 min. The maximal (uncoupled) rate was measured via the addition of FCCP and the nonmitochondrial oxygen consumption analyzed via the addition of antimycin A (n = 3, three runs/experiment). (B) Complex I–NADH: Ubiquinone reductase activity from *gba*^*+/+*^, *gba*^*+/−*^, and *gba*^*−/−*^ brains expressed as a ratio to citrate synthase (n = 3). (C) Complex II–III: Succinate dehydeogenase, cytochome *c* reductase activity from *gba*^*+/+*^, *gba*^*+/−*^, and *gba*^*−/−*^ brains expressed as a ratio to citrate synthase (n = 3). (D) Complex IV activity from *gba*^*+/+*^, *gba*^*+/−*^, and *gba*^*−/−*^ brains expressed as a ratio to citrate synthase (n = 3). (E) Neurons were treated with 3 nM decylTPP and MitoQ_10_ for 48 hr and stained with TMRM and fluorescence intensity analyzed via confocal microscopy (n = 3, >16 cells/experiment). (F) *gba*^*+/+*^and *gba*^*−/−*^ cells treated with 3 nM decylTPP and MitoQ_10_ for 48 hr were subjected to immunoblotting using the indicted antibodies. All data in this figure represent the mean ± SEM, ^*^p < 0.05.

Mitochondrial oxygen consumption was measured in an attempt to account for the bioenergetic defect. Basal, maximal, and minimal oxygen consumption rates were measured by additions of oligomycin (ATPase inhibitor), FCCP (uncoupler to show maximal respiration), and antimycin A (to show nonmitochondrial oxygen consumption) (Figure 4A and Table S2). Oligomycin inhibited respiration coupled to oxidative phosphorylation in control cells but not in *gba*^*−/−*^ cells. FCCP accelerated respiration to maximum capacity in both *gba*^*+/+*^ and *gba*^*+/−*^ cells but to a lesser extent in *gba*^*−/−*^ cells. These observations suggest a generalized defect in the respiratory chain. Therefore, complex I (CI), CII-III, and CIV activity assays were performed (Figures 4B–4D and Table S3) and data normalized to citrate synthase activity, as mitochondrial accumulation had been observed in the *gba*^*−/−*^ neurons (Figure S3A). CI and CII-III levels were significantly reduced between *gba*^*+/+*^/*gba*^*+/−*^ and *gba*^*−/−*^ (Figures 4A and 4B). CIV activity was not significantly different across any genotype (Figure 4C). Steady-state levels of components of CI-V were comparable between *gba*^*+/+*^, *gba*^*+/−*^, and *gba*^*−/−*^ following SDS-PAGE analysis (Figure S3B), as was the complex V regulatory protein, ATPIF1 (Figure S3C). Assembly of CI, CII, and CV in *gba*^*−/−*^ cells, as assessed on BN-PAGE, was not affected compared to controls (Figure S3D). In an attempt to elucidate the cause of the mitochondrial dysfunction in *gba*^*−/−*^ cells, MitoQ_10_ was employed (Kelso et al., 2001). MitoQ_10_ is a mitochondrial targeted antioxidant (through the lipophilic triphenylphosphonium cation) and is thought to offset the effects of inner membrane lipid peroxidation by inhibiting generation of reactive oxygen species (ROS) from CI (Murphy and Smith, 2007). Prior to confocal imaging with TMRM to analyze ΔΨ_m_, midbrain neurons were treated with 3 nM MitoQ_10_ and the control compound decylTPP (structurally similar but lacking the ubiquinone moiety of MitoQ_10_) for 48 hr (Figure 4E). Although MitoQ_10_ had a negligible effect on ΔΨ_m_ of *gba*^*+/+*^ and *gba*^*+/−*^ neurons, it significantly increased the ΔΨ_m_ of *gba*^*−/−*^ midbrain neurons to the point at which the mitochondrial potential was no longer substantially different from that of controls. LC3 levels of *gba*^*+/+*^ and *gba*^*−/−*^ cells treated with decylTPP and MitoQ_10_ were analyzed via immunoblotting; MitoQ_10_ treatment did not increase either LC3I/II levels, suggesting mitochondrial dysfunction is a secondary event to defective autophagy (Figure 4F). Taken together, these data strongly suggest a functional respiratory chain defect in *gba*^*−/−*^ mitochondria, possibly though generation of ROS via CI.

### *gba*^*−/−*^ Mitochondria Are Fragmented with Increased Levels of S-OPA1 Isoform

**Figure 5:**
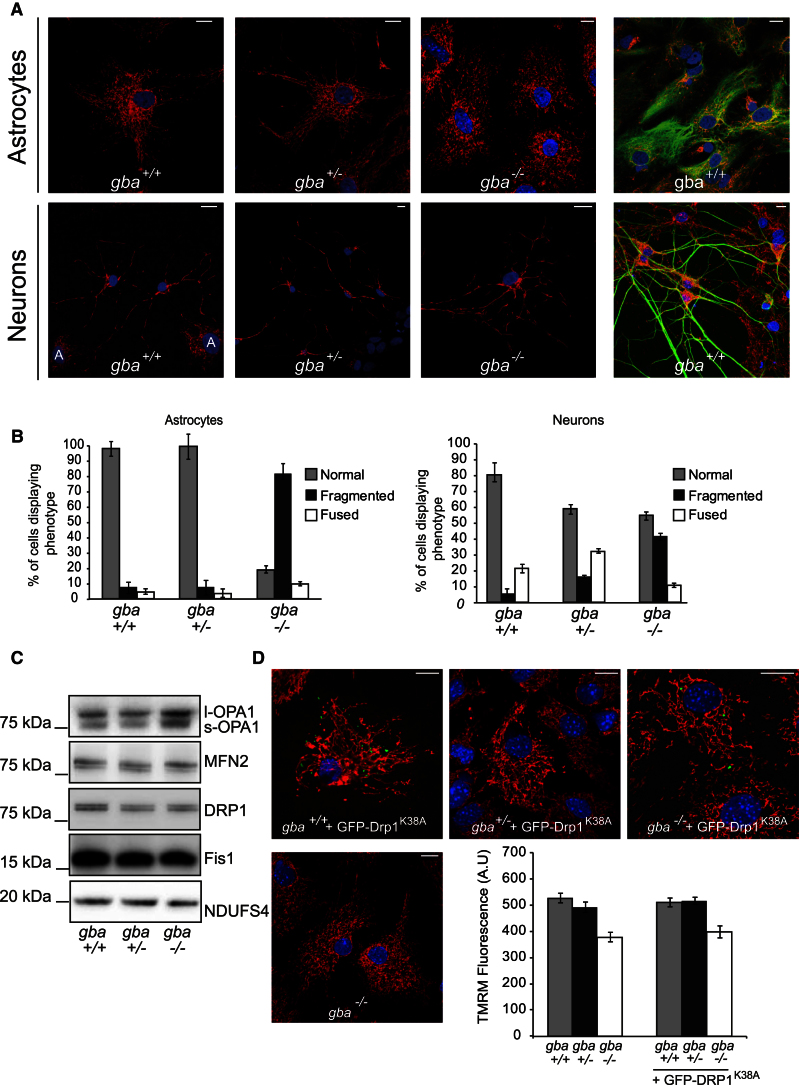
Mitochondrial Morphology Is Affected in *gba*^*−/−*^ Cells (A) Neurons and astrocytes were immunostained for cytochrome *c* and stained with Hoechst. Far left panels were additionally immunostained for GFAP and MAP2 to mark neurons and astrocytes. “A” in nucleus denotes astrocytes. Scale bar, 20 μm. (B) Mitochondrial morphology was blind counted from cells in (A) with neurons scored as MAP2 positive and astrocytes as GFAP positive. A “normal” morphology is classified as a mixture of fused and fragmented mitochondria that comprise a wild-type network. (n = 3; 100 cells counted/experiment). (C) Levels of morphology proteins from *gba*^*+/+*^, *gba*^*+/−*^, and *gba*^*−/−*^ isolated mitochondria were analyzed via immunoblotting with the indicated antibodies. (D) Midbrain *gba*^*+/+*^, *gba*^*+/−*^, and *gba*^*−/−*^ astrocytes expressing GFP-DRP1^K38A^ were immunostained for cytochrome *c* and mitochondrial morphologies analyzed; concurrently cells were stained with TMRM and fluorescence intensity analyzed via confocal microscopy. Scale bar, 20 μm (n = 3, >15 cells/experiment). All data in this figure represent the mean ± SEM.

Increased mitochondrial fragmentation can suggest dysfunction and is a prerequisite for removal of damaged mitochondria via autophagy (Twig et al., 2008). Confocal imaging of midbrain neurons and astrocytes showed mitochondrial fragmentation in *gba*^*−/−*^ cells, in comparison to *gba*^*+/+*^ and *gba*^*+/−*^ cells (Figure 5A). While the mitochondrial fragmentation was more evident in astrocytes, *gba*^*−/−*^ neuronal mitochondria were also fragmented compared to those in *gba*^*+/+*^ and *gba*^*+/−*^ (Figure 5B). Western blot analysis revealed no changes in levels of mitochondrial shaping proteins Drp1 or Mfn2 in *gba*^*+/+*^, *gba*^*+/−*^, or *gba*^*−/−*^ cells (Figure 5C). However levels of the short isoform of optic atrophy 1 (S-OPA1) were increased in the *gba*^*−/−*^ cells. To understand whether the aberrant mitochondrial morphology was cause or consequence of the impaired mitochondrial bioenergetics, *gba*^*+/+*^, *gba*^*+/−*^, and *gba*^*−/−*^ astrocytes were transfected with the dominant-negative fission mediator GFP-Drp1^K38A^ to rescue the fragmented network (Figure 5D). As expected, *gba*^*+/+*^ control cells expressing GFP-Drp1^K38A^ exhibited a fused network indicating a block of mitochondrial fission, the same phenotype was observed in the *gba*^*+/−*^ cells (Figure 5D). Expression of GFP-Drp1^K38A^ in *gba*^*−/−*^ cells partially restored the mitochondrial network to a wild-type state, although the network did not appear completely restored (Figure 5D). Although mitochondria in *gba*^*−/−*^ cells appeared to be fusion competent, rescue of the mitochondrial network did not extend to ΔΨ_m_. *gba*^*+/+*^, *gba*^*+/−*^, and *gba*^*−/−*^ astrocytes transfected with GFP-Drp1^K38A^ were incubated with TMRM and ΔΨ_m_ assayed via confocal microscopy (Figure 5D). Relative TMRM fluorescence was not altered by expression of GFP-Drp1^K38A^. Inability of GFP-Drp1^K38A^ to rescue ΔΨ_m_ suggests that the fragmented mitochondrial morphology displayed by *gba*^*−/−*^ cells is a consequence and not a cause of impaired mitochondrial bioenergetic function.

### Defective Mitophagy in *gba*^*−/−*^ Neurons and Astrocytes

**Figure 6:**
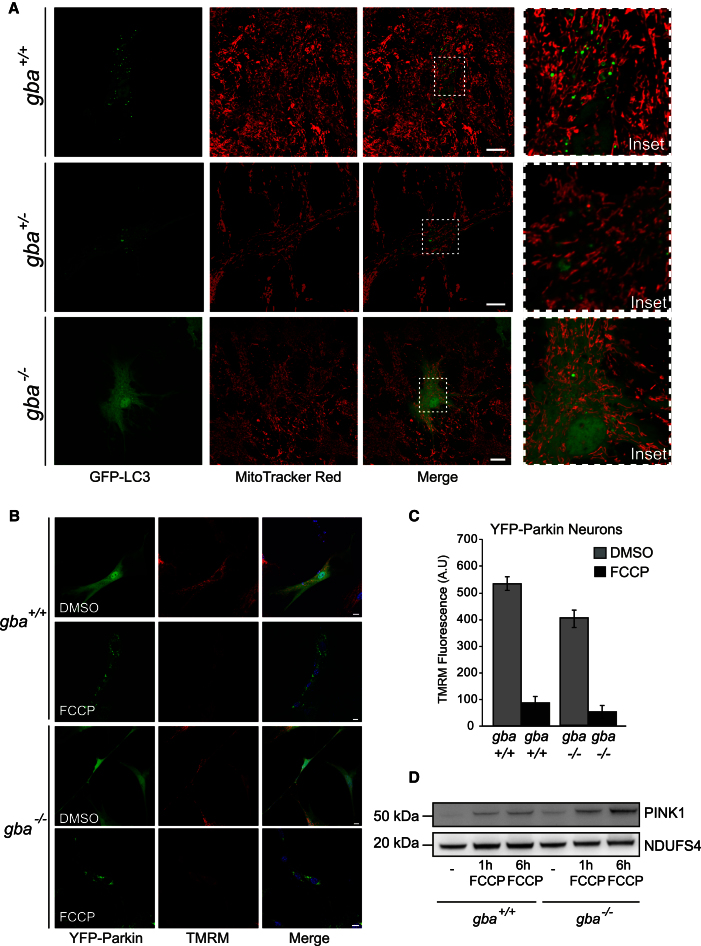
Impaired Mitophagy in *gba*^*−/−*^ Neurons and Astrocytes (A) Midbrain astrocytes were transfected with GFP-LC3, treated with 100 nM bafilomycin A1, and stained with MitoTracker Red and imaged using confocal microscopy. (B) Midbrain *gba*^*+/+*^ and *gba*^*−/−*^ neurons and astrocytes were transfected with YFP-Parkin. Half the transfected cells were treated for 1 hr with 10 μM FCCP and all bathed in TMRM containing recording solution and imaged using confocal microscopy. Scale bar, 20 μm. (C) TMRM fluorescence intensity of cells from (A) was analyzed via confocal microscopy. Data represent the mean ± SEM, (n = 3, >4 cells analyzed per experiment). (D) Mitochondria within midbrain neurons were uncoupled by the addition of 10 μM FCCP for 1 and 6 hr. PINK1 expression analyzed via immunoblotting on isolated mitochondria from *gba*^*+/+*^ and *gba*^*−/−*^ neurons. Mitochondrial CI subunit NDUFS4 was used as a loading control.

To determine whether the mitophagy pathway in *gba*^*−/−*^ cells was functional, astrocytes were transfected with GFP-LC3, subsequently treated with 100 nM bafilomycin A1 for 8 hr, and stained with MitoTracker Red. Formation of autophagosomes and autophagosome/mitochondrial colocalization were evident in control astrocytes (Figure 6A, upper two panels). However, few GFP-LC3 labeled autophagosomes were seen in *gba*^*−/−*^ astrocytes, in which the majority of GFP-LC3 was in the cytosolic/LC3I form (Figure 6A, lower panel). Thus, there was no colocalization of autophagosomes with mitochondria in gba^*−/−*^ cells. In order to address whether mitochondria in *gba*^*−/−*^ cells were marked for mitophagy, yet not degraded due to defects upstream in the autophagy pathway, Parkin recruitment assays were performed. Recruitment of the cytosolic E3-ubiqutin ligase Parkin to its mitochondrial outer membrane receptor PINK1 is a necessary step in mitophagy. In cells with a negligible ΔΨ_m_, PINK1 cannot be imported to the mitochondrial inner membrane and localizes to the outer membrane (trapped at the TOM complex), where it recruits Parkin, flagging dysfunctional mitochondria for turnover (Jin et al., 2010; Lazarou et al., 2012). *gba*^*+/+*^ and *gba*^*−/−*^ neurons were transfected with YFP-Parkin and treated with or without 10 μM FCCP for 1 hr at 37°C and then loaded with 25 nM TMRM for 30 min. As with untreated control cells, *gba*^*−/−*^ cells failed to recruit YFP-Parkin, despite a reduced ΔΨ_m_ (Figures 6B and 6C). Recruitment of Parkin was only observed upon FCCP treatment and complete dissipation of ΔΨ_m_. To ensure this inability to recruit Parkin was not due to reduced levels of PINK1 at mitochondria, cells were uncoupled with FCCP prior to mitochondrial isolation to promote accumulation of PINK1 on the mitochondrial outer membrane (Figure 6C). Once depolarized, there was no disparity in levels of PINK1 in *gba*^*−/−*^ neurons in comparison with controls. This confirms that although mitochondria are dysfunctional, ΔΨ_m_ in *gba*^*−/−*^ neurons is not low enough to recruit Parkin, which was only recruited to the mitochondrial surface when the ΔΨ_m_ was completely dissipated with an uncoupler. These damaged mitochondria thus remain unmarked for turnover via mitophagy and so accumulate in *gba*^*−/−*^ neurons.

## Discussion

Neuronopathic (type II) Gaucher disease is a rapidly progressive neurodegenerative disease leading to cognitive impairment and a shortened life expectancy. The data presented here argue that the neurodegenerative process underlying this disease results from downregulated autophagy and an impaired proteasomal system. As a result, dysfunctional mitochondria, ubiquitinated proteins, and insoluble α-synuclein accumulate, culminating in an impaired bioenergetic state. Coupled with an increase in apoptotic markers and cell death in various brain regions including the hippocampus, cortex, and cerebellum (Enquist et al., 2007), these data suggest a mechanism for severely compromised neuronal viability that follows the loss of GCase.

While defects in autophagy have been reported in other LSDs, the specific role of impaired autophagy in the pathogenesis of neurodegeneration in GD is not clear (Sun and Grabowski, 2010). This is interesting in light of recent findings suggesting a genetic link between *gba* mutations and PD, and the role of several gene products associated with familial PD in mitophagy. In addition, it has been suggested that LSDs could be broadly classified as diseases of autophagy (Settembre et al., 2008b). The failed clearance of damaged organelles is associated with neurodegeneration in both multiple sulphatase deficiency and mucopolysaccharidosis type IIIA and IV (de Pablo-Latorre et al., 2012; Jennings et al., 2006; Settembre et al., 2008a). In these models, damaged and fragmented mitochondria accumulated within cells and displayed a reduced capacity to buffer calcium, leading to increased susceptibility to cell death (Jennings et al., 2006; Settembre et al., 2008a).

Much attention has recently been paid to links between autophagy and the UPS in neurodegenerative disease (Korolchuk et al., 2009). α-synuclein aggregates are observed in PD and PD-GBA brains, and it is therefore of interest that p62/SQSTM1 and α-synuclein accumulation is seen in the GD mouse brain. The lysosomal degradation pathway is primarily responsible for the clearance of α-synuclein intermediate species, although there is mounting evidence for a role of the proteasome (Webb et al., 2003). α-synuclein species are trafficked to the lysosome via CMA (Cuervo et al., 2004). Defects in this pathway have been implicated in familial forms of PD, where mutated α-synuclein is able to bind to the lysosomal receptor LAMP-2A but unable to cross into the lumen resulting in a block of CMA (Cuervo et al., 2004). Furthermore, it has been reported that depletion of GCase results in a bidirectional feedback loop in which α-synuclein accumulates and a causes self-propagating disease (Gegg et al., 2012; Mazzulli et al., 2011). Indeed, in the mouse model of type II GD we observed α-synuclein accumulation. Furthermore, we have recently shown that inhibition of GCase by conduritol-β-epoxide, or knockdown of *gba*, also induces changes in mitochondrial function and increased α-synuclein levels (Cleeter et al., 2013). Thus, GD appears to demonstrate the disruption of proteostasis by specific protein aggregation.

Mitochondrial dysfunction has been shown to play a central role in PD and related neurodegenerative conditions (Schapira, 2007). Many of the features of mitochondrial dysfunction we have described in *gba*^*−/−*^ cells are mirrored in familial PD models. A reduction in mitochondrial membrane potential, basal and maximal oxygen consumption, and reversal of the ATPase was described in PINK1 KO neurons (Gandhi et al., 2009). However, in this model ΔΨ_m_ could be rescued by the addition of CI and CII substrates. This was not the case in GD, suggesting that the defect lies primarily within the respiratory chain. Although there are strong associations between reduced CI activity and PD, it is not seen uniformly, making it complicated to delineate (del Hoyo et al., 2010; Schapira et al., 1990a; Wood-Kaczmar et al., 2008). Our data suggest that in type II GD, CI is fully assembled into the holoenzyme, yet activity is reduced. It is difficult to establish whether CI damage reported in PD patients is mediated by the same mechanisms as in GD, as the mechanisms by which CI defects cause neurodegeneration remain elusive (Schapira, 2010). One theory is that damage occurs due to generation of ROS from CI and CIII. CI, at nearly 1 MDa in size, is the largest complex of the respiratory chain and most susceptible to damage by oxidative stress (Lin and Beal, 2006). Incidentally, Lewy body disease, which is considered by many to be a preclinical indictor of PD, has been shown to have intermediate levels of CI activity, further implicating mitochondrial dysfunction in neurodegeneration (Schapira et al., 1990b). In addition to reduced CI activity, we also found reductions in CII/CIII activity. A decrease in CIII function has been reported in lymphocytes and platelets from PD patients, with reduced activity ascribed to ROS generation via increased electron leakage from CIII (Haas et al., 1995). It is interesting to note that the same pattern of respiratory chain defect (CI-CIII) is seen in Freidreich’s ataxia (thought to relate to iron-sulfur cluster defects) and is associated with free radical damage (Bradley et al., 2000). As this is the first reported study of mitochondrial dysfunction in GD, further investigation is required to determine whether there are respiratory chain defects in postmortem GD patients and if they mirror those in PD.

Regulation of mitochondrial shape is essential for cell homeostasis (Chan, 2006). Fragmented mitochondria with diminished ΔΨ_m_ show reduced fusion capacity and, under physiological conditions, are removed by autophagosomal sequestration (Twig et al., 2008). Mitochondria observed in this model of type II GD were fragmented, but were not removed due to defects in quality control pathways. Although altered mitochondrial morphology has been implicated in many neurodegenerative diseases (Chen et al., 2007; Palmer et al., 2011), this loss of normal morphology did not appear to be the primary cause of cellular dysfunction in GD neurons, but rather a secondary effect of upstream cellular stress. The fact that mitochondrial fragmentation in *gba*^*−/−*^ neurons could be rescued by dominant-negative Drp1 suggests that mitochondria retained fusion competence. This may be due to the fact that there is still a large proportion of L-OPA1 isoform responsible for inner membrane fusion (Ishihara et al., 2006).

Fragmented and damaged mitochondria are normally removed from the population via mitophagy, a process initiated by Parkin recruitment following the accumulation of PINK1 on the outer mitochondrial membrane in response to a fall in ΔΨ_m_ (Narendra et al., 2008; Twig et al., 2008). In *gba*^*−/−*^ neurons the potential is not low enough for Parkin recruitment and only occurred when ΔΨ_m_ was fully dissipated with FCCP. Thus mitochondria in type II GD, while severely dysfunctional, are not flagged for turnover through the PINK1/parkin mitophagy pathway and so accumulate within the cells. This does raise the point, however, that within a cell, even one harboring as severe a disease as type II GD, for mitochondria to be flagged for turnover by the PINK1/Parkin pathway, ΔΨ_m_ must be near completely dissipated. Whether this occurs physiologically in vivo remains to be seen. These observations also illustrate the importance of the reversal of the ATPase which in these cells prevented collapse of ΔΨ_m_, and so serves to maintain a population of dysfunctional mitochondria which should otherwise be removed.

**Figure 7:**
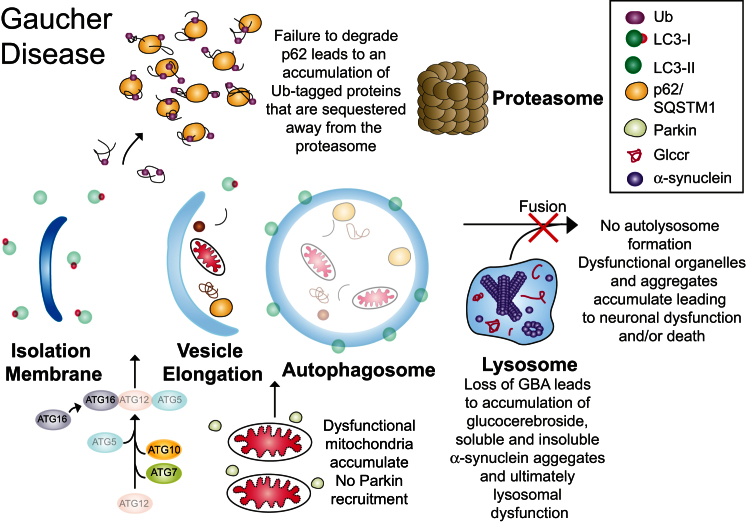
Dysfunctional Quality Control in Gaucher Disease With loss of *gba*, lysosome function is severely impaired, and autophagy does not occur. Dysfunctional organelles accumulate, including mitochondria. Due to loss of *gba* and the self-propagation of α-synuclein, the autophagic pathway is disrupted, compromising UPS function. The damaged mitochondrial respiratory chain cannot support the potential, which decreases to a level at which the ATPase reverses. A decreased ΔΨ_m_ limits their ability to re-enter the fission-fusion cycle; this low ΔΨ_m_, maintained by the ATPase, appears to be sufficient in maintaining a potential above the critical level required to recruit Parkin, and thus mitochondria are not flagged for turnover, amplifying the defect.

In summary, we have shown that primary neurons and astrocytes from a mouse model of neuronopathic GD harbor dysfunctional mitochondria that accumulate in cells due to defects in both protein and organellar degradation machinery. This is schematized in Figure 7. It must be emphasized, however, that type II GD is a severe form of the disease and that our mice die at P15. It would be of interest to determine the impact on the phenotype of the neuronopathic GD mice of an approach that could improve the mitochondrial bioenergetic status or that could upregulate autophagy in vivo. As α-synuclein deposits were only observed in the intact brains of young mice and the phenotype observed in the mice is already severe in the neonate as early as P1, this would require treatment of the fetuses in utero. Any further work needed to investigate subtleties in aged mice would need to be undertaken on the *gba*^*+/−*^ genotype, or those with mutations that mimic those in patients.

It appears that the pathogenesis of neurodegeneration in both type II GD and in PD stems from defects in proteostasis. The end result is a toxic buildup of protein aggregates and dysfunctional organelles until a pathogenic threshold is reached, resulting in neuronal cell death (Enquist et al., 2007). These insights into neurodegenerative pathways that result from defective autophagy may signpost the links between *gba* mutations and PD and help to illuminate pathophysiological mechanisms in PD and potentially provide novel targets for therapy (Schapira and Gegg, 2013).

## Experimental Procedures

### Cell Culture, Transfection, and Live-Cell Imaging

Mixed cultures of hippocampal and midbrain neurons and glial cells (neurons >70% of culture population) were prepared as described previously (Abramov et al., 2004), with some modifications. A detailed description of the isolation can be found the Supplemental Experimental Procedures. Cells were cultured and maintained in Neurobasal media (Invitrogen) supplemented with B-27 (Invitrogen), penicillin/streptomycin (Sigma), 10% fetal bovine serum (GIBCO), and 2 mM L-glutamine (Sigma). Cultures were maintained at 37°C in a humidified atmosphere of 5% CO_2_ and 95% air. Media was replaced every 5 days and maintained for a minimum of 10 days before experimental use to ensure the expression of receptors. Cells were used 10–14 days postisolation for all experiments. *gba*^*+/+*^, *gba*^*+/−*^, and *gba*^*−/−*^ mixed cultures were always compared to litter mates. After 10 days, cells were transfected with either GFP-LC3, GFP-Drp1^K38A^, or YFP-Parkin constructs using Lipofectamine LTX with plus reagent (Invitrogen) according to the manufacturer’s instructions. For live-cell imaging, cells were stained with 25 nM tetramethyl rhodamine methyl ester (TMRM) (Invitrogen) in HBSS for 30 min at room temperature. To observe changes in ΔΨ_m_, oligomycin (1 μM) and FCCP (1 μM) (Sigma) were added to cells while imaging, while 5 mM methyl pyruvate and 10 mM methyl succinate (Sigma) were added 5 min prior to imaging. To visualize YFP-Parkin recruitment, transfected cells were either treated with 10 μM FCCP or DMSO for 1 hr at 37°C. To visualize autophagosome accumulation in astrocytes transfected with GFP-LC3, cells were treated with 100 nM bafilomycin for 8 hr at 37°C and stained with 50 nM MitoTracker Red CMX*Ros* (Molecular Probes). To measure mitochondrial mass, cells were stained with 1 μM calcein-AM (Molecular Probes) and 50 nM MitoTracker Red CMX*Ros* for 20 min at 37°C. Neurons were treated with 3 nM MitoQ and 3 nM decylTPP (control compound) (M. Murphy, Cambridge) for 48 hr and stained with TMRM to measure ΔΨ_m._

### Immunofluorescence

For immunofluorescence assays, cells 14 days postisolation were fixed with 3.7% (w/v) paraformaldehyde (Sigma) and permeabilized with 0.2% Triton X-100 with 3% BSA in PBS. Cells were stained with anti-cytochrome *c*, anti-p62/SQSTM1 (BD Biosciences), and anti-MAP2 and anti-GFAP (Abcam) primary antibodies and were visualized using mouse-Alexa Fluor 555 (Invitrogen) or rabbit/chicken-FITC 488 (Sigma). Nuclei were stained with 10 μg/mL Hoechst 33258 (Sigma).

### Brain Stem Histology

Sagittal brain stem sections were stained using the automated Discover XT staining module (Ventana Medical Systems). Briefly, sections were dewaxed and treated with Protease 3 (Ventana). Sections were stained with α-synuclein (Abcam) and swine anti-rabbit antibodies (Dako). DAP Map kit (Ventana) was used for subsequent detection. Hematoxylin and bluing agents (Ventana) were added as counterstains.

### Microscopy

Confocal microscopy was conducted using a Zeiss LSM 700 confocal microscope and 40× and 63× oil-immersion objectives. For Z sectioning the size of slices collected was 0.1 μm. TMRM, MitoTracker Red CMX*Ros*, and Alexa Fluor 555 were excited using the 543 nm laser line (HeNe laser) and fluorescence measured using a 560 nm long-pass filter. Calcien-AM and FITC-488 were excited using the 488 laser line (Argon laser) and the fluorescence measured using a 530 nm band pass filter. Hoechst 33258 (Sigma) staining was detected with the 405 nm diode laser. Images were captured using Zeiss Zen software. Images were processed using ImageJ (http://rsbweb.nih.gov/ij/index.html), Zeiss, and/or MetaMorph (Visitron Systems) software.

### Western Blotting

Cell extracts and brain tissue were separated into whole-cell and mitochondrial fractions using differential centrifugation. Briefly, for whole-cell isolation, cells were solubilized in RIPA buffer (150 mM NaCl, 0.5% sodium deoxycholate, 0.1% SDS, 1% TX-100, 50 mM Tris [pH 8.0], 1 mM PMSF). A detailed description of mitochondrial isolation can be found in the Supplemental Experimental Procedures. Protein concentration was determined using a bicinchoninic acid kit (Pierce). SDS-PAGE protein extracts were prepared in 2× lamelli sample buffer (Bio-Rad) with β-mercaptoethanol (Sigma) and 30–50 μg separated on either 10% or 12% Bis-Tris NuPAGE (Novex). Antibodies used for western blotting in this study are detailed in the Supplemental Experimental Procedures.

### Autophagy and UPS Assays

To monitor autophagic flux, cultured midbrain neurons were either treated with 100 nM bafilomycin A1 (Sigma), 1 μM rapamycin (Stress Marq Biosciences), or both drugs in combination for 8 hr at 37°C. Alternately, cells were serum and amino acid starved in EBSS (Earle’s balanced salt solution) (Invitrogen) for 16 hr at 37°C. Cell extracts were isolated in RIPA as above. For proteasomal activity assays, midbrain neurons were treated with either 10 μM MG132 (Cayman Chemical) or DMSO for 8 hr at 37°C. Cell extracts were isolated in RIPA as above. Chymotypsin-like activity of the proteasome was measured using the Proteasome-Glo Chymotrypsin-like cell-based assay (Promega). Briefly 15,000 cells per well were seeded onto white-bottomed 96-well plates. Half the wells were treated with 10 μM MG132 and the other vehicle DMSO control for 8 hr at 37°C. Chymotypsin-Glo buffer and substrate were added directly to cells and luminesce measured after 30 min in a FLUOstar Optima plate reader (BMG Labtech).

### α-Synuclein Aggregation Assays

Dissected midbrain sections were resuspended and homogenized in 5 volumes of ice-cold TX-100 lysis buffer (50 mM Tris, 175 mM NaCl, 5 mM EDTA, 1% TX-100, 1 mM PMSF), briefly sonicated, and incubated on ice for 30 min. Samples were centrifuged at 14,000 *g* for 15 min at 4°C and the resulting supernatant deemed the soluble α-synuclein fraction. The pellet (insoluble fraction) was resuspended in lysis buffer containing 2% SDS and 8 M urea. This fraction was not boiled prior to western analysis.

### Respiratory Chain Enzymatic Assays

Complex I (NADH:Ubiquinone reductase), complex II–III (succinate, cytochrome *c* reductase), and complex IV (cytochrome oxidase) from P1 mice brains were determined as previously described (Oppenheim et al., 2009). All activities are expressed as a ratio to citrate activity to account for mitochondrial enrichment.

### Statistical Analysis

Data were generated from a minimum of three independent experiments. Littermates were compared and constituted one independent experiment. Statistical analysis was performed using Microsoft Xcel and Minitab software. For all graphs and traces, error bars represent the mean ± SEM. For Figure 2A we used Minitab Software to apply a one-way ANOVA, with Tukey posthoc comparison. For Figures 3B–3D we used Minitab Software to apply a General Linear Model where the two factors were genotype and brain region. We used a Tukey posthoc pairwise comparison with 95% confidence intervals. All other data where possible and appropriate were analyzed using a two-tailed Student’s t test. All significance was expressed as ^*^p < 0.05.

## Supplemental Information

Document S1. Figure S1, Figure S2, Figure S3, Table S1, Table S2, Table S3, Supplemental Experimental Procedures, and Supplemental References
