# Supplementary material for: Mitochondria and Quality Control Defects in a Mouse Model of Gaucher Disease—Links to Parkinson’s Disease
Source: Cell Metab. 2013 Jun 4;17(6):941–53. doi: 10.1016/j.cmet.2013.04.014 (PMC3678026; doi:10.1016/j.cmet.2013.04.014)
Supplement: Document S1. Figure S1, Figure S2, Figure S3, Table S1, Table S2, Table S3, Supplemental Experimental Procedures, and Supplemental References [file mmc1.pdf]

Cell Metabolism, Volume *17*

Supplemental Information

Mitochondria and Quality Control Defects in a Mouse Model of Gaucher Disease—Links to Parkinson's Disease

Laura D. Osellame, Ahad A. Rahim, Iain P. Hargreaves, Matthew E. Gegg, Angela Richard-Londt, Sebastian Brandner, Simon N. Waddington, Anthony H.V. Schapira, and Michael R. Duchen

SUPPLEMENTAL INFORMATION INVENTORY

1. Supplemental Figures S1-S3
2. Legends of Figures S1-S3
3. Supplemental Tables S1-S3
4. Supplemental Experimental Procedures
5. Supplemental References

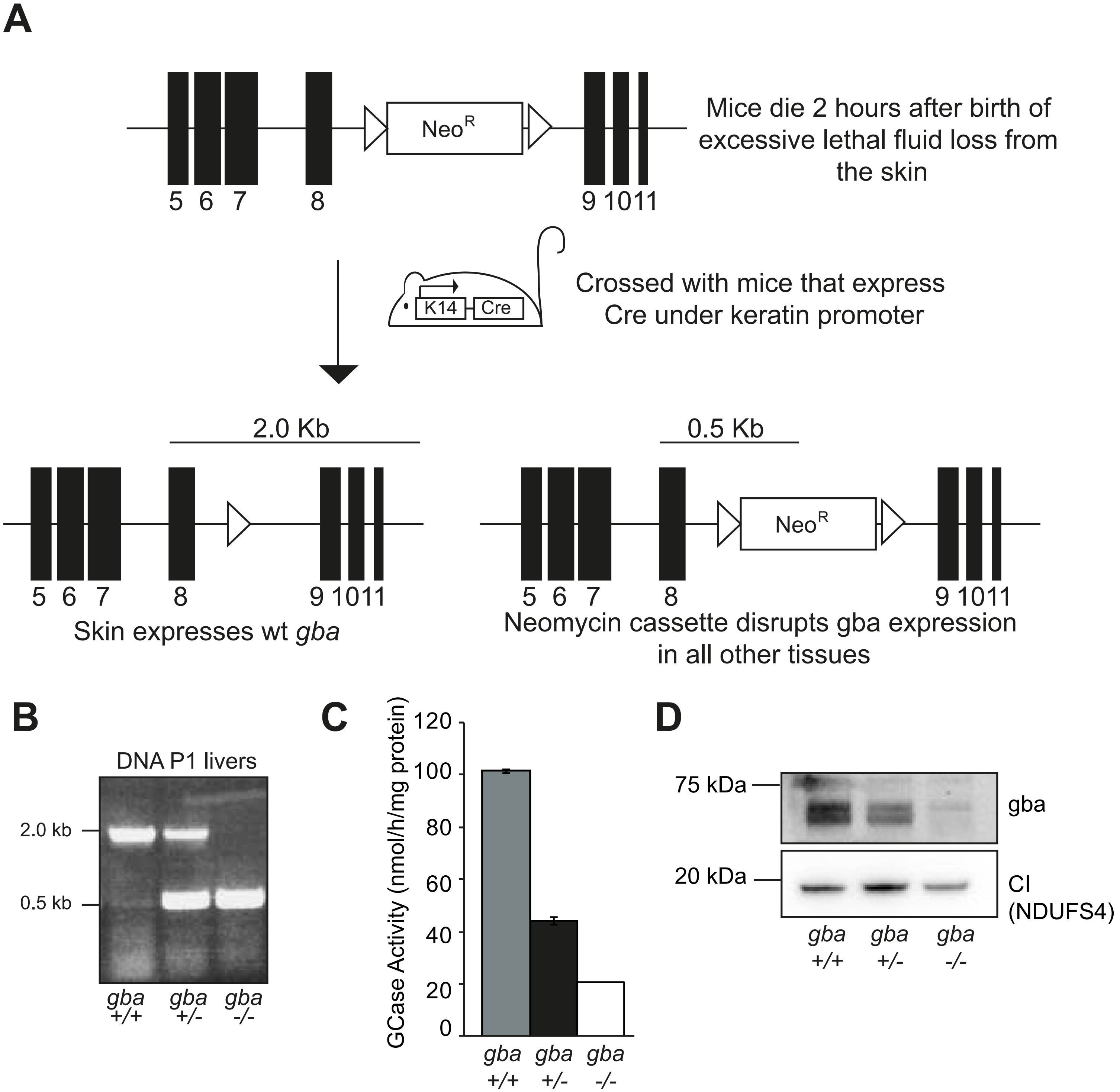

Sup Figure S.1 Osellame et al

Figure S1. ***gba* expression levels.** (A) Schematic of mouse model. Whole body knockout of mouse *gba* is lethal two hours after birth as the animal dies not from GD but from excessive epidermal fluid loss. *gba* KO mice were crossed with K14 mice expressing Cre recombinase under a keratin promoter allowing Cre mediated removal of the neomycin cassette in the skin only. Mice named in Enquist et al as K14-wt, K14-lnl/wt (lox/neomycin/lox) and K14-lnl/lnl will hereby be referred to as $gba^{+/+}$, $gba^{+/-}$, $gba^{-/-}$ respectively. (B) Genotyping from P1 mice. cDNA isolated from liver and resulting PCR products analyzed by agarose electrophoresis (C) GCase activity monitored in cortical neurons and astrocytes. (D) gba protein levels from $gba^{+/+}$, $gba^{+/-}$ and $gba^{-/-}$ midbrain analyzed via immunobloting using gba antibodies. Mitochondrial CI subunit NDUFS4 was used as a loading control.

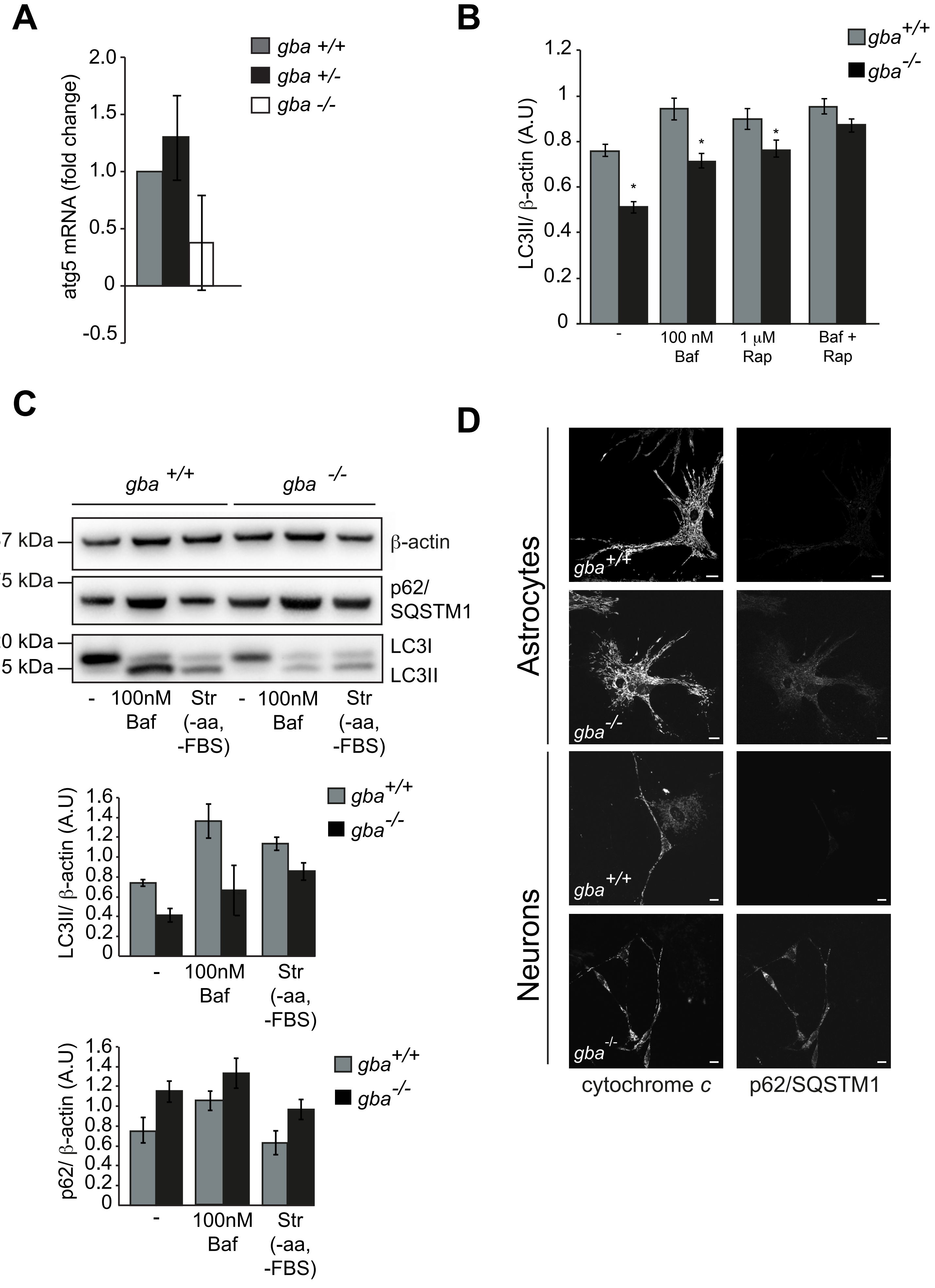

Sup Figure S.2 Osellame et al

Figure S2. **Accumulation of mitochondrial mass though inhibition of autophagy and UPS.** Related to **Figure 1.** (A) qPCR of m*Atg5* expressed as a fold change using the ΔC_T method. Mouse *TBP* (TATA binding protein) was used as a reference gene. Data represents the mean ± SEM (n=3, triplicate samples per condition and genotype) *p values <0.05. (B) Densitometry analyzes of autophagy of (Figure 1C) expressed as a ratio of LC3II/β-actin. (C) p62/SQSTM1 and LC3I/II levels analyzed in response to starvation (EBSS-amino acids and FBS). β-actin was used as a loading control. LC3II and p62/SQSTM1 were analyzed via densitometry and expressed as a ratio over β-actin. (D) $gba^{+/+}$ and $gba^{-/-}$ midbrain neurons and astrocytes were immunostained cytochrome *c* (mitochondria) for p62/SQSTM1. Scale bar = 20 μm.

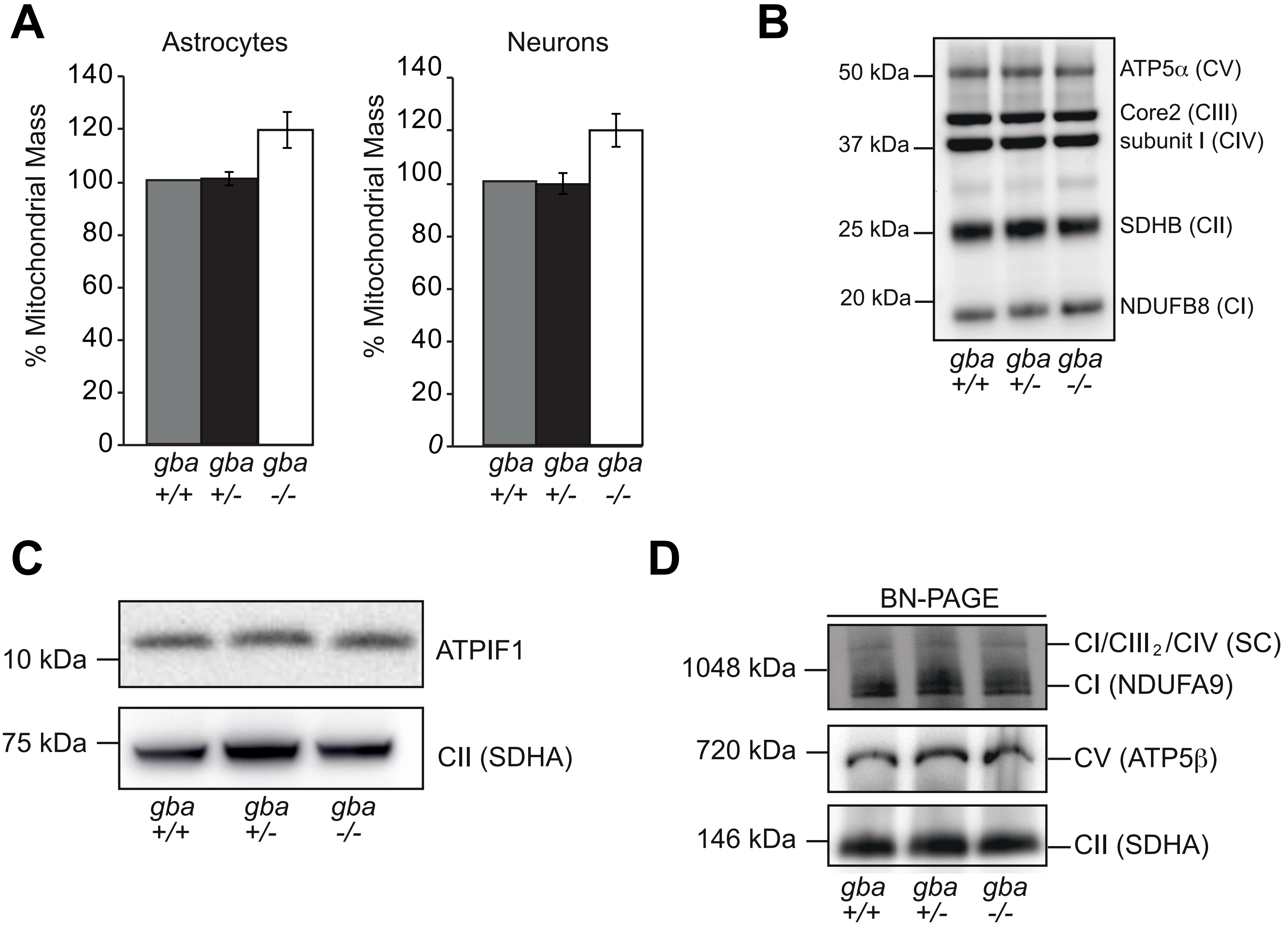

Sup Figure S.3 Osellame et al

Figure S3. **Assembly of the mitochondrial respiratory chain is unaffected in *gba*$^{-/-}$ neurons and astrocytes.** Related to **Figure 3 and 4.** (A) Cortical neurons and astrocytes were stained with Calcein-AM and MitoTracker Red. Resulting images were binerized and mitochondrial mass calculated as a percentage of cytosol volume. Data represents the mean ± SEM, (n=3, > 4 cells analyzed per experiment). (B) Levels of mitochondrial respiratory chain proteins were analyzed via immunobloting using a total OXPHOS cocktail antibody. (C) ATPIF1 protein levels from *gba*$^{+/+}$, *gba*$^{+/-}$ and *gba*$^{-/-}$ midbrain analyzed via western blotting. Mitochondrial complex II subunit SDHA was used as a loading control. (D) Assembly of CI, CII and CV from *gba*$^{+/+}$, *gba*$^{+/-}$ and *gba*$^{-/-}$ mitochondria analyzed using Blue-Native PAGE and immunoblotted using the indicated antibodies.

SUPPLEMENTAL TABLES

Table S1. ***gba* enzyme activity (nmol/h/mg protein)** Related to **Figure S1.** GCase activity rates from isolated hippocampal neurons.

	$gba^{+/+}$	$gba^{+/-}$	$gba^{-/-}$
GCase activity (nmol/h/mg protein)	101.5 ± 0.9	43 ± 1.9	26.65

Table S2. **Mitochondrial Oxygen Consumption Rates (nmol/O^2/min/10^6 cells).** Related to **Figure 3.** Mitochondrial oxygen consumption rates from isolated neuronal midbrain cultures were measured in a Clark-type electrode using oligomycin (inhibitor of CV), FCCP (to measure maximal oxygen consumption rate) and antimycin A (as a measure of non-mitochondrial oxygen consumption). Data represents the mean ± SEM, (n=3, 3 runs / experiment).

	$gba^{+/+}$	$gba^{+/-}$	$gba^{-/-}$
**Basal**	0.99 ± 0.41	0.94 ± 0.34	0.57 ± 0.25
**Oligomycin**	0.51 ± 0.27	0.73 ± 0.43	0.54 ± 0.15
**FCCP (Maximal)**	3.96 ± 0.11	3.8 ± 0.13	1.6 ± 0.74
**Antimycin A**	0.10 ± 0.18	0.12 ± 0.1	0.12 ± 0.13

Table S3. **Mitochondrial Respiratory Chain Activities.** Related to **Figure 3.** Mitochondrial respiratory chain activities from whole brain (CI, CII-CIII and CIV) expressed as a ratio to citrate synthase were measured in *gba*$^{+/+}$, *gba*$^{+/-}$ and *gba*$^{-/-}$. Data represents the mean ± SEM, (n=3)

	gba$^{+/+}$	***gba***$^{+/-}$	***gba***$^{-/-}$
**CI**	0.158 ± 0.001	0.143 ± 0.009	0.108 ± 0.01
**CII-CIII**	0.133 ± 0.015	0.115 ± 0.009	0.081 ± 0.009
**CIV**	0.018 ± 0.004	0.014 ± 0.003	0.016 ± 0.026

SUPPLEMENTAL EXPERIMENTAL PROCEEDURES

Generation of Transgenic Mice. *gba* mice were generated by Enquist et al., and a detailed description of generation, primers and sequencing procedures can be found there (Enquist et al., 2007). For this study heterozygous breeding pairs were employed. P1-P3 mice were genotyped using liver cDNA isolated using the QIAGEN DNeasy blood and tissue kit (Invitrogen) according to manufacturer's instructions. Animal husbandry and experimental procedures were performed in full compliance with the UK Animal (Scientific Procedures) Act of 1986.

Isolation of neurons and astrocytes: Hippocampal and midbrain sections were dissected and placed in ice-cold HEPES-buffered salt solution (HBSS) (Sigma). Tissue was minced and digested in 0.05% Trypsin (GIBCO) for eight minutes at 37˚C, pelleted and washed repeatedly in HBSS. The tissue was resuspended in DMEM and sheared by passage through a narrow gauge syringe and plated onto Poly L-lysine coated 22mm glass coverslips for imaging or dishes for western blotting.

GCase activity assays: GCase activity assays were preformed as previously described (Gegg et al., 2012). Briefly, hippocampal neurons were homogenized in buffer containing 250 nM sucrose, 10 mM Tris (pH 7.4), 1 mM ethylenediametetraacetic acid, PMSF, 1 μg/mL pepstatin A, 1 μg/mL leupeptin and 1 mM sodium orthovanadate. Homogenate was diluted to 2 mg/mL in water, sonicated and gba activity was measured using 20 μg of protein by hydrolysis of 5 mM methylumbelliferyl-β-D-glucopyranoside in McIlvaine

buffer (pH 5.4) with 22 mM sodium taurocholate at 37˚C for 1 hour. An addition of 250 mM glycine (pH 10.4) inhibited the reaction and 4-methylumbelliferone fluorescence was measured (excitation 365 nm / emission 450 nm).

qPCR: RNA was isolated from cultured neurons and astroctyes derived from *gba*$^{+/+}$, *gba*$^{+/-}$ and *gba*$^{-/-}$ mice, using the QIAGEN RNeasy kit (Invitrogen) according to manufactures instructions. 1 μg of total RNA was used to generate cDNA using SuperScript II Reverse Transcriptase (Invitrogen). The following primer sequences were used to amplify mouse *Atg5* (Forward: AGAAGCAGAACACTACTATT, Reverse: AAGATGTTAGTGAGATATGGT) and mouse *TBP* (TATA binding protein) (Forward:GCAACAACAGCAGGCAGTA, Reverse: TGGTGTGGCAGGAGTGAT). SYBR Green JumpStart (Sigma) was used to measure transcript levels in a C1000 Thermal Cycler with a CFX96 RT platform (Biorad). qPCR analysis (ΔC_T method) was performed using the CFX Manager software (Biorad).

Mitochondrial Isolation: Tissue or cells resuspended in mitochondrial isolation buffer (20 mM HEPES, 220 mM mannitol, 70 mM sucrose, 1 mM EDTA, 1 mM PMSF, pH 7.6) were homogenized using a drill-fitted pestle and clarified at 800 *g* (Johnston et al., 2002). Brain tissue was subjected to several rounds of homogenization and differential centrifugation for mitochondrial isolation. Samples were pelleted at 10,000 *g* yielding a crude mitochondrial fraction. An additional clarifying spin at 800 *g* was performed prior to the final 10,000 *g* spin resulting in an enriched mitochondrial fraction. Protein

concentration was determined using a bicinchoninic acid (BCA) kit (Pierce Thermo Fisher).

Oxygen Consumption. Mitochondrial oxygen consumption was analyzed using an Oxytherm Clarke type electrode. Briefly two million cells (mixed midbrain neurons and astrocytes) were placed in the chamber thermostatically maintained at 37˚C. The electrode was calibrated with oxygen saturated water, assuming 406 oxygen atoms / mL water at 37˚C. The basal oxygen consumption rate of cells was recorded in respiration media (HBSS with 4.2 mM $NaHCO_3$). Maximal oxygen consumption rates were obtained using 4 µM oligomycin and 2 µM FCCP at defined 2 minute intervals. To observe non-mitochondrial oxygen consumption 2 µM antimycin A was added at completion of the experiment.

Antibodies used in western blotting. The following primary antibodies were used for western blotting: anti-LC3b (MBL), anti-ATP5β (Abcam), anti-GBA (CalBiochem), anti-Ubiquitin, anti-K48 Ubiquitin, anti-K63 Ubiquitin (Cell Signaling), anti-total OXPHOS cocktail, anti-NDUFS4, anti-NDUFA9, anti-SDHB (Mitosciences, Invitrogen) anti-β-actin, anti-Mitofusin 2, anti-Atg5/12 (Sigma), anti-α-synuclein, anti-OPA1, anti-p62/SQSTM1, anti-cytochrome *c* and anti-DRP1 (BD Biosciences). ECL chemiluminescent substrate (GE Healthcare) was used to detect immunoreactive proteins on blots.

Blue-Native Polyarcylamide Gel Electrophoresis (BN-PAGE). Mitochondrial extracts were prepared for Novex precast BN-PAGE according to manufacturer's instructions. Briefly, 20 μg of purified mitochondria were solubilized in 1% digitonin buffer (Invitrogen) and Coomassie blue G loading buffer (Invitrogen) added prior to separation on 4-12% native gels. Gels were transferred to activated PVDF and detected as above.

SUPPLEMENTAL REFERENCES

Enquist, I.B., Lo Bianco, C., Ooka, A., Nilsson, E., Mansson, J.E., Ehinger, M., Richter, J., Brady, R.O., Kirik, D., and Karlsson, S. (2007). Murine models of acute neuronopathic Gaucher disease. Proc. Natl. Acad. Sci. USA *104*, 17483-17488.

Gegg, M.E., Burke, D., Heales, S.J., Cooper, J.M., Hardy, J., Wood, N.W., and Schapira, A.H. (2012). Glucocerebrosidase deficiency in substantia nigra of parkinson disease brains. Ann. Neurol. *72*, 455-463.

Johnston, A.J., Hoogenraad, J., Dougan, D.A., Truscott, K.N., Yano, M., Mori, M., Hoogenraad, N.J., and Ryan, M.T. (2002). Insertion and assembly of human tom7 into the preprotein translocase complex of the outer mitochondrial membrane. J. Biol. Chem. *277*, 42197-42204.
